# Ureteropelvic junction obstruction in infants: Open or minimally invasive surgery? A systematic review and meta-analysis

**DOI:** 10.3389/fped.2022.1052440

**Published:** 2022-11-23

**Authors:** Valentina Cascini, Giuseppe Lauriti, Dacia Di Renzo, Maria Enrica Miscia, Gabriele Lisi

**Affiliations:** ^1^ Pediatric Surgery Unit, “Spirito Santo” Hospital of Pescara, Pescara, Italy; ^2^ Department of Medicine and Aging Science, “G. d'Annunzio” University of Chieti-Pescara, Chieti, Italy; ^3^ Pediatric Surgery Unit, “Spirito Santo” Hospital of Pescara, Pescara, Italy

**Keywords:** ureteropelvic junction obstruction, Anderson–Hynes dismembered pyeloplasty, minimally invasive surgery, infants, systematic review, meta-analysis

## Abstract

**Introduction:**

The historical gold standard treatment for ureteropelvic junction obstruction (UPJO) was the open Anderson–Hynes dismembered pyeloplasty (OP). Minimally invasive surgery (MIS) procedures, including laparoscopic pyeloplasty (LP) and robot-assisted laparoscopic pyeloplasty (RALP), have been reported to achieve better outcomes (i.e., decreased morbidity, reduced postoperative pain, superior esthetic results, and shortened length of hospital stay, LOS), with a success rate similar to OP. The main limitation of the MIS approach is the age and weight of patients, limiting these procedures to children >1 year. This study aims to evaluate the feasibility and benefits of MIS pyeloplasty compared to OP to surgically treat UPJO in children <1 year of age.

**Materials and methods:**

A systematic review was independently performed by two authors. Papers comparing both techniques (MIS pyeloplasty vs. OP) in infants were included in the meta-analysis. Data (mean ± DS or percentage) were analyzed using Rev.Man 5.4 A *p *< 0.05 was considered significant.

**Results:**

Nine studies (eight retrospective and one prospective) meet the inclusion criteria. A total of 3,145 pyeloplasties have been included, with 2,859 (90.9%) OP and 286 (9.1%) MIS. Age at operation was 4.9 ± 1.4 months in OP vs. 5.8 ± 2.2 months in MIS, *p* = ns. Weight at surgery was 6.4 ± 1.4 kg in OP vs. 6.9 ± 1.4 kg in MIS, *p* = ns. Operative time was 129.4 ± 24.1 min for OP vs. 144.0 ± 32.3 min for MIS, *p* < 0.001. LOS was 3.2 ± 1.9 days for OP vs. 2.2 ± 0.9 days for MIS, *p* < 0.01. Postoperative complications were present in 10.0 ± 12.9% of OP vs. 10.9 ± 11.6% in MIS, *p* = ns. Failure of surgery was 5.2 ± 3.5% for OP vs. 4.2 ± 3.3% for MIS, *p* = ns.

**Conclusion:**

The development of miniaturized instruments and technical modifications has made MIS feasible and safe in infants and small children. MIS presented a longer operative time than OP. However, MIS seemed effective for treating UPJO in infants, showing shortened LOS compared to OP. No differences have been reported with regard to the incidence of postoperative complications and failure of pyeloplasty. Given the low quality of evidence of the meta-analysis according to the GRADE methodology, we would suggest limiting MIS procedures in infants to only those high-volume centers with experienced surgeons.

## Introduction

The historical gold standard for the treatment of pediatric ureteropelvic junction obstruction (UPJO) was the open Anderson–Hynes dismembered pyeloplasty (OP), with a reported success rate of 90%–100% (1).

Over the last decades, minimally invasive surgery (MIS) procedures, i.e., conventional laparoscopic pyeloplasty (LAP) and robot-assisted laparoscopic pyeloplasty (RALP), have been reported to be a possible replacement for OP (2). Few reports and a recent meta-analysis have confirmed that both LAP and RALP seemed safe and effective (3–7). Moreover, a multicenter study comparing both approaches in pediatrics has confirmed how both procedures were safe and as successful as OP, with an incidence of failure of <5% (2). Moreover, LAP and RALP have been reported to correlate with several advantages, such as decreased morbidity, reduced postoperative pain, superior esthetic results, and shortened length of hospital stay (LOS). The main limit to the MIS approach has been reported to depend on the age and weight of the patients, limiting these procedures to children >1 year (1, 8). Since the publication of Tan’s work in 1999 (9), where LAP was not recommended in small children, there has been a doubt about performing LAP in small children. The main concerns were related to the operative field offered by a pneumoperitoneum in infants, the limited space for port placement, the small working space, and the small ureteral diameter. However, following studies have established that LAP was safe and feasible in infants (10, 11).

More recently, RALP seemed to have advantages of maneuverability, improved vision, comfort in suturing, and improved ergonomics compared to LAP (4, 6). The main issues in RALP are the absence of correct-sized trocars for infants and the shortage of robots in most pediatric units because of their cost (2, 8). It has been reported how MIS pyeloplasty was extremely uncommon in infants, even if the incidence of RALP procedures has boosted over the last years (12). Following an increased knowledge of MIS pyeloplasty, there have been few publications on LAP and RALP in infants over the last few years. However, most of them have reported outcomes in a reduced number of cases. RALP has been reported to simplify the MIS approach in children,, with results comparable to OP (13). However, to the best of our knowledge, only a few studies were focused on infants. Those comparing the results of the different approaches (OP vs. MIS) were extremely scarce. In the present study, we aimed to compare the outcomes of OP vs. MIS (both LAP and RALP) in infants affected by UPJO.

## Materials and methods

### Data sources and study selection

The present study was registered on PROSPERO (registration # CRD42022358981), an international database of prospectively registered systematic reviews (National Institute for Health Research) (14). The systematic review was drafted according to the Preferred Reporting Items for Systematic Reviews and Meta-Analyses (PRISMA) statement (15).

Using a stated search strategy (Table 1), two investigators (VC, GLa) individually screened the main databases (PubMed/Medline, Scopus, Web of Science, and Cochrane) with combined keywords. MeSH headings and terms used were “Pyeloplasty” AND “Infants” (Supplementary material S1). Studies published from 1984 to August 2022 in English language were included. The list of references was screened as well to detect possible pertinent cross references. Case reports, opinion articles, and reviews were excluded. All comparative studies reporting the outcomes of OP compared to those of MIS (i.e., LAP and/or RALP) to treat UPJO in infants or patients <15 kg were included. The full text of theoretically suitable papers was retrieved and individually assessed for eligibility by the same two authors. Any divergence over the entitlement of papers was solved through a further debate with a third author (GLi).

**Table 1:** Inclusion criteria of the systematic review.

Publication	
Language	English
Time period	January 1984–August 2022
Subject	Human studies
Study type	Retrospective
	Prospective
	Case–control
	Cohort
Excluded	Case report
	Case series (<10 patients)
	Editorials
	Letters
	Gray literature
Keywords	Pyeloplasty
	Infants

The studies comparing OP vs. MIS for the Anderson–Hynes pyeloplasty in infants were included in the meta-analysis. The exclusion criteria are as follows:

• treatment other than Anderson–Hynes pyeloplasty, retroperitoneoscopy, OTAP, recurrent UPJO, secondary UPJO, patients >1 year; and
• studies without valid data about the comparison of these two techniques.

### Data analysis

Categorical variable rates were compared with Pearson's *χ*^2^ test or two-tailed Fisher’s exact probability test. When median and range were reported, mean ± SD were valued (16).

The meta-analysis was managed with RevMan 5.4 (17). The random effects model was selected. The risk ratio (RR) was assessed for categorical variables. Differently, mean differences (MD) were preferred in the case of continuous variables. Both results were reported with 95% confidence intervals (CIs). Data were expressed as mean ± SD. *I*^2^ values were used to judge homogeneity and quantify the dispersion of effect sizes. Biases among the papers included were evaluated with the funnel plot. Quantitative and demographic data were compared using Fisher's exact test and expressed as number, percentage, or mean ± SD using the RR and 95% CI. A *p *< 0.05 was considered significant.

### Quality assessment

Two authors (DDR and MEM) assessed the risk of bias for individual studies. This assessment was achieved with a methodological index for nonrandomized studies (MINORS) (18). Dissimilarities between the two authors (DDR and MEM) were solved through a discussion with a third author (GLi). The score for this index ranges between 0 and 24 points. The “gold standard” cutoff was 19.8 points. With regard to the quality of each outcome, we graded the quality of evidence, thanks to the Grading of Recommendations Assessment, Development and Evaluation (GRADE) methodology (19). The quality of evidence was graded as high, moderate, low, and very low in all results. Observational studies were assessed as low quality of evidence. The quality of evidence was further reduced in the case of risk of bias, inconsistency, indirectness imprecision, and publication bias. MINORS was adopted to judge the risk of bias in observational papers. Inconsistency was determined according to heterogeneity, and *I*^2^ value was used to evaluate heterogeneity. As established in Cochrane guidelines, heterogeneity was assessed as low, moderate, substantial, and considerable when *I*^2^ values were 0–40, 30–60, 50–90, and 75%–100%, respectively (20). If a score overlapped two groups, we inserted a mixed inconsistency (e.g., low/moderate) in our GRADE table. Finally, imprecision was evaluated with optimal information size (OIS) based on 25% relative risk reduction, 0.05 a-error, and 0.20 b-error (21).

## Results

### Systematic review

**Figure 1:**
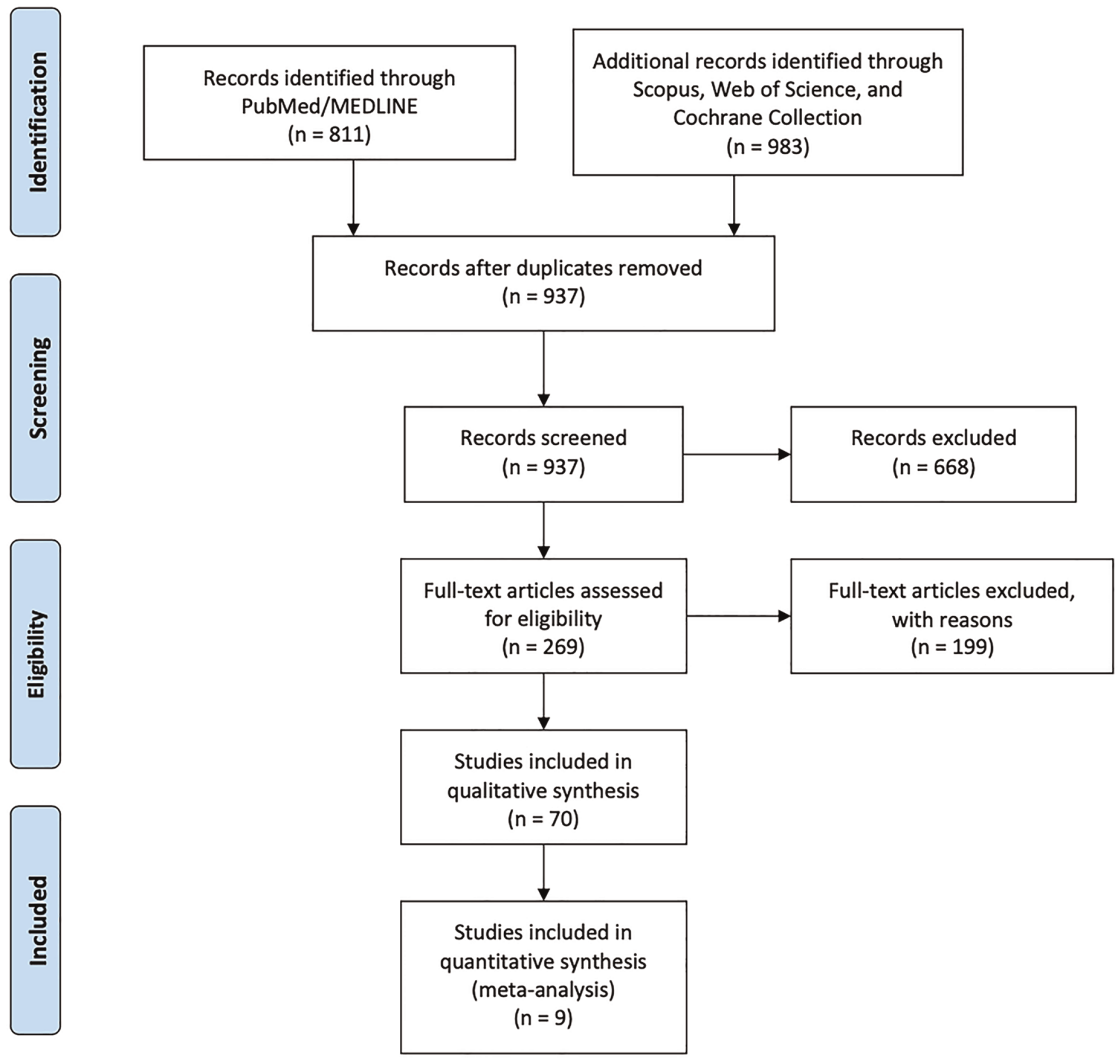
Diagram of workflow in the systematic review and meta-analysis.

The initial review retrieved 811 studies from databases using keywords “Pyeloplasty” AND “Infants.” Thanks to the screening of all these titles and abstracts, we selected 70 papers focusing on infants or children <15 kg of weight with UPJO. Among these, only 13 publications were comparative studies between OP vs. MIS (1, 13, 22–32) (Figure 1).

Six studies (1, 22, 24, 25, 27, 31) described OP vs. LAP, and one was excluded (31) due to incomplete data. Three papers treating OP vs. RALP (26, 30, 32) were included. Two studies compared OP vs. LAP + RALP (13, 28); one was excluded (28) due to incomplete information. Two papers (23, 29) treating RALP vs. LAP were excluded.

### Meta-analysis

Nine articles were included in the meta-analysis, eight retrospective studies (1, 22, 24–27, 30, 32) and one prospective study (13).

The total number of pyeloplasties performed in infants and patients <15 kg of weight was 3,145, with 2,859 (90.9%) OP and 286 (9.1%) MIS, comprising 145 (50.7%) LAP and 141 (49.3%) RALP. The M/F ratio was 3:1, with 2,301M (73.2%) and 844F (26.8%). Of 2,859 OP patients, 2,092 were males (73.2%) and 767 were females (26.8%); of 286 in the MIS group, 209 were males (73.1%) and 77 were females (26.9%), with no differences between OP and MIS groups [*p* = ns, RR: 1.001, 95% CI: (0.93–1.08), Table 2].

**Table 2:** Demographic data of papers included in the meta-analysis.

	OP	MIS	*p*-Value
M (%)	2,092/2,859 (73.2)	209/286 (73.1)	ns*
F (%)	767/2,859 (26.8)	77/286 (26.9)	
Left kidney (%)	162/279 (58.1)	101/183 (55.2)	ns*
Right kidney (%)	117/279 (41.9)	82/183 (44.8)	
Age (months)	4.9 ± 1.4	5.8 ± 2.2	ns
Weight (kg)	6.4 ± 1.4	6.9 ± 1.4	ns
Follow-up (months)	21.5 ± 8.1	13.9 ± 4.7	ns

OP, open pyeloplasty; MIS, minimally invasive surgery.

*Fisher's exact test.

The side of the kidney affected by UPJO has been reported in seven papers (1, 22, 24–26, 30, 32), with 462 patients (279 OP and 183 MIS). Of 279 OP patients, 162 had left kidneys affected (58.1%) and 117 had right kidneys affected (41.9%). Of 183 in the MIS group, 101 had left kidneys affected (55.2%) and 82 had right renal units affected (45%), with no differences between the two groups [*p* = ns, RR: 1.052, 95% CI (0.89–1.24), Table 2].

**Figure 2:**
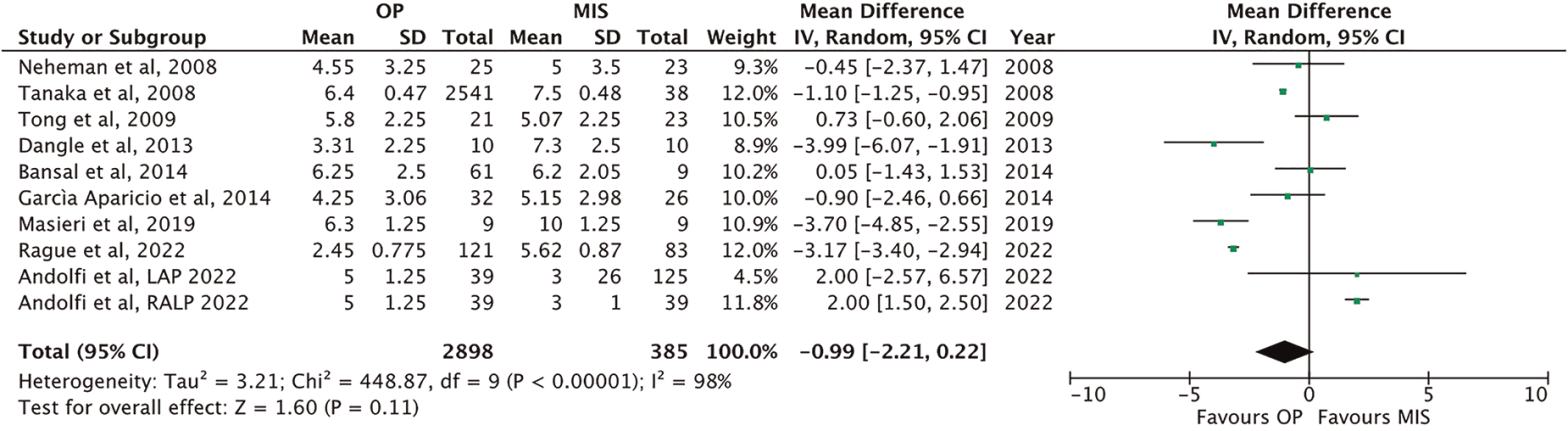
Forest plot comparison of age at procedure between OP and MIS in infants.

The mean age at procedure has been reported in nine papers (1, 13, 22, 24–27, 30, 32), with no difference between OP infants (4.9 ± 1.4 months) and MIS patients [5.8 ± 2.2 months; *p* = ns, MD −0.9, 95% CI: (−2.21–0.22), Table 2 and Figure 2].

**Figure 3:**
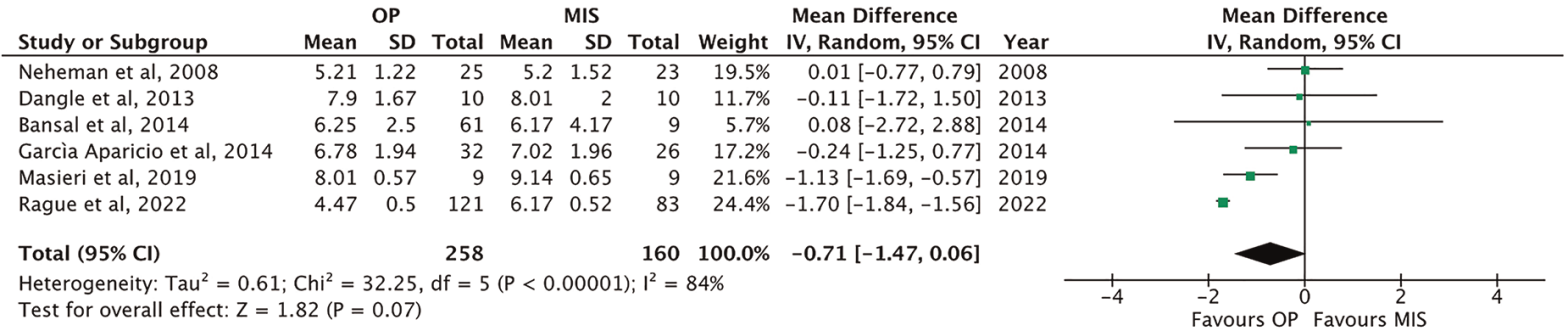
Forest plot comparison of weight at procedure between OP and MIS in infants.

The weight at surgery has been reported in six of the included papers (1, 22, 24, 26, 30, 32). Weight was not significantly different among the two groups: 6.4 ± 1.4 kg in OP vs. 6.9 ± 1.4 in MIS [*p* = ns, MD −0.71, 95% CI (−1.47–0.06), Table 2 and Figure 3].

**Figure 4:**
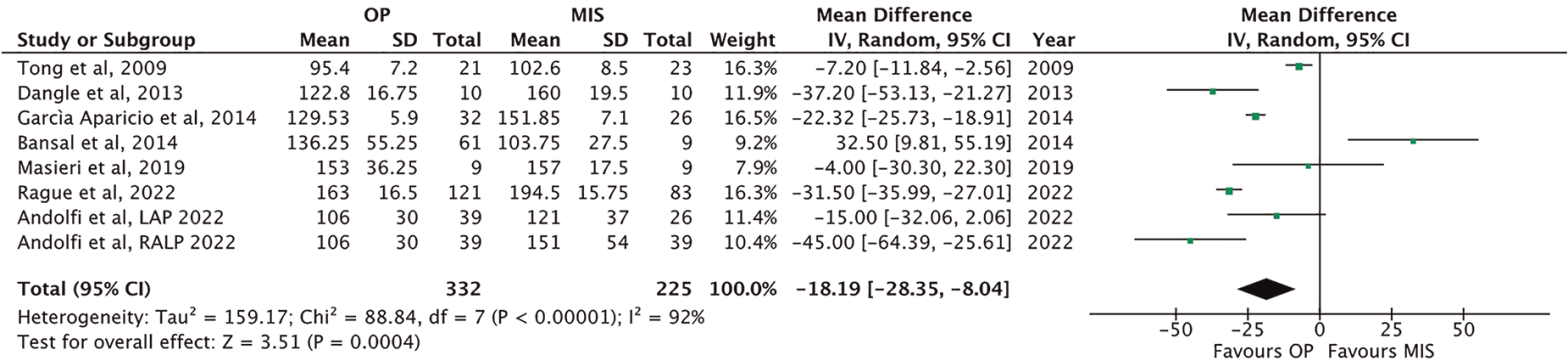
Forest plot comparison of operative time at procedure between OP and MIS in infants.

Operative time (OT) has been reported in seven studies (1, 24, 26, 32, 25, 13, 32). OT was significantly lower in OP than that in MIS [129.4 ± 24.1 vs. 144.0 ± 32.3 min, respectively; *p* = 0.0004, MD: −18.19, 95% CI: (−28.35, −8.04), Figure 4].

**Figure 5:**
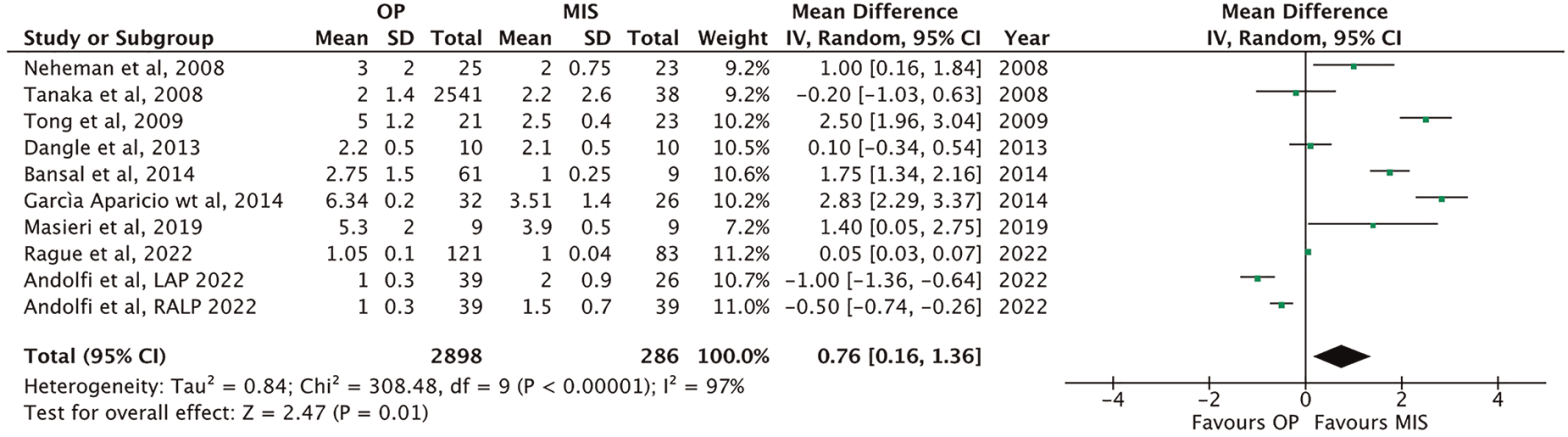
Forest plot comparison of the length of hospital stay between OP and MIS in infants.

Nine papers (1, 13, 22, 24–27, 30, 32) have shown an increased LOS in OP compared to that in MIS [3.2 ± 1.9 vs. 2.2 ± 0.9 days, respectively; *p* = 0.01, MD: 0.76, 95% CI: (0.16–1.36), Figure 5].

**Figure 6:**
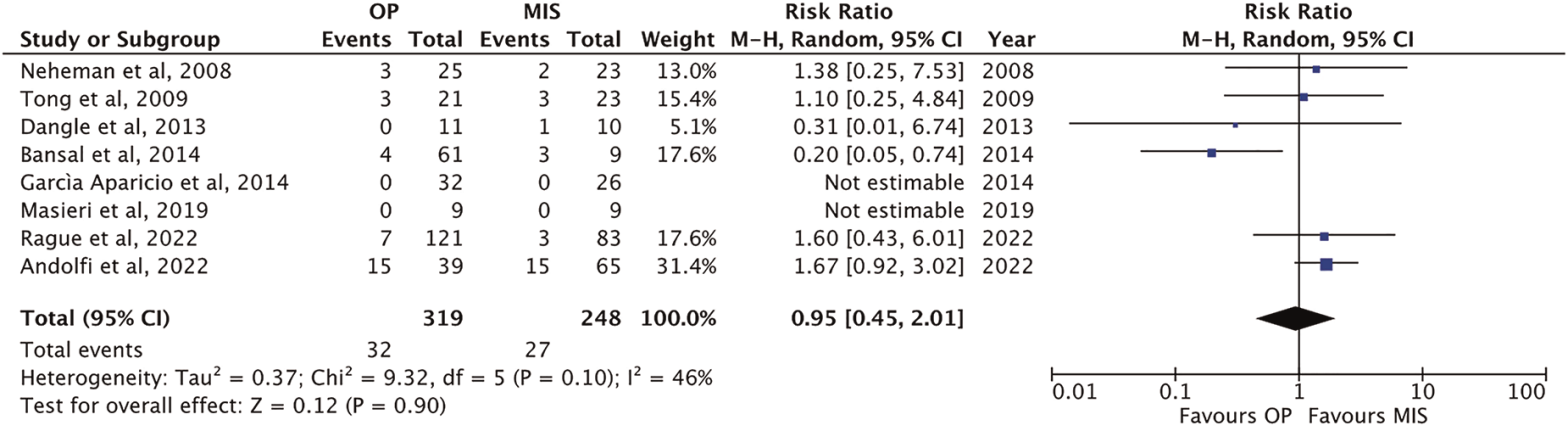
Forest plot comparison of the incidence of postoperative complications between OP and MIS in infants**.**

Eight studies have been reported on postoperative complications (1, 13, 22, 24–26, 30, 32), such as urinary leakage, urinary infection, and bleeding. The incidence of complications was comparable between the two groups: 10.0 ± 12.9% in OP (32/319 patients) vs. 10.9 ± 11.6% in MIS procedures [27/248 patients; *p* = ns, RR: 0.95, 95% CI (0.45–2.01), Figure 6].

**Figure 7:**
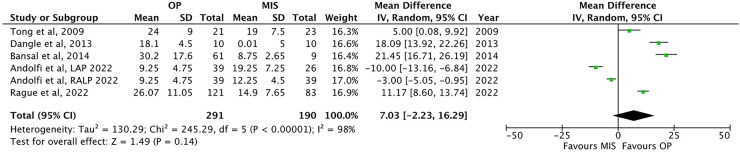
Forest plot comparison of the length of follow-up between OP and MIS in infants.

The length of postoperative follow-up has been reported in five papers (13, 25, 26, 30, 32). OP presented a longer but not significant follow-up than MIS [21.5 ± 8.1 months vs. 13.9 ± 4.7 months, respectively; *p* = ns; MD: 7.03, 95% CI: (−2.23–16.29), Table 2 and Figure 7].

**Figure 8:**
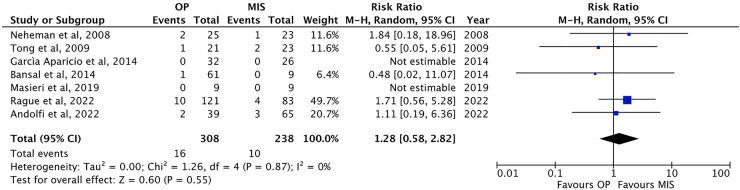
Forest plot comparison of the incidence of failure between OP and MIS in infants.

The failure rate has been mentioned in seven papers (1, 13, 22, 24–26, 30). In most of the studies included, success has been defined as resolutions of symptoms and improved ultrasonographic or renographic parameters at the follow-up. The failure rate was not different in OP (5.2 ± 3.5%, 16/308 patients) and MIS [4.2 ± 3.3%, 10/238 patients; *p* = ns; RR: 1.28, 95% CI: (0.58–2.82), Figure 8].

Only the paper by Andolfi et al. (13) further compared LAP vs. RALP. No conversion was reported in both groups. Furthermore, the incidence of complications and the success rates were similar for both procedures.

We further screened these nine papers included in the meta-analysis with regard to the outcomes between left surgery and right surgery. However, no data were reported on operative time, the length of hospital stay, the incidence of complications, and the failure rate regarding the side of the procedure.

## Discussion

The indications to surgically treat UPJO in infants are specific, including impaired renal function, severe hydronephrosis causing a mass effect, recurrent urinary tract infection (UTI), worsening of hydronephrosis with thinning of renal parenchyma, or UPJO in a solitary kidney (8, 30, 33).

The gold standard procedure to treat UPJO is the Anderson–Hynes dismembered pyeloplasty, commonly performed through an open miniflank approach in infants (13, 33–36). MIS procedures have progressively been adopted over the last years, although these procedures are technically challenging with the need for a long learning curve, especially for LAP. However, these procedures seemed safe and effective, as an increasing number of studies have reported similar outcomes compared to OP in pediatric patients. Several meta-analyses comparing OP and MIS showed that children in the MIS group were older than those in OP, with the same success rate and complications for both techniques (3, 5, 6). MIS procedures have shown benefits in terms of shortened LOS, decreased postoperative pain, and enhanced esthetic outcomes in older pediatric cases, gaining increased popularity as an alternative to OP (2, 37).

However, MIS was preferred in older children, with infants still receiving OP (6, 38, 39). Liu et al. indicated in the Kid's Inpatient Database (KID) that age is the only characteristic that augmented the odds of having MIS (40). In infants, the miniflank lumbotomy is preferred for several reasons. First, it avoids muscle splitting, thus decreasing postoperative pain and allowing a fast recovery. Second, it allows for a direct approach to the posterior side of both the renal pelvis and the ureter (8). A cohort study including a nationwide inpatient sample data (time period 2008–2010) has reported that the distribution of OP and MIS approaches for pyeloplasty in infants was about 78% and 0.7%, respectively (28). The limits of the utilization of MIS pyeloplasties in infants or smaller children depend on the technical aspects unique to this population. First, the increase of intra-abdominal pressure and the peritoneal absorption of CO_2_ due to the pneumoperitoneum could bring physiological and time-depending respiratory issues, such as displacement of the diaphragm and acidosis (23, 41). A pressure of 10 mmHg or greater may cause a reduction in venous return, right ventricular output, left cardiac output, and bradycardia due to vagal reflex. When greater than 8 mmHg, the pneumoperitoneum could cause renal issues because of the stimulation of the renin–angiotensin–aldosterone system, with consequent emission of the antidiuretic hormone, leading to salt and water retention with oliguria (41). The further concern in this population is the limited space for port placement and the restricted working space, making the procedure challenging (11, 42).

While LAP showed a lengthier learning curve and known technical difficulties, RALP has become more accepted because of instruments with 7 degrees of motion, a 3D screen with magnification, and hand-tremor reduction. Moreover, it has been reported that the learning curve for RALP seemed to be comparable to the one for OP (4, 8). The current availability of miniaturized instruments has improved the use of MIS. In LAP, a 5-mm camera and 3-mm instruments enhance the ability to perform the anastomosis, reporting similar results to OP (10, 24). Regarding RALP, the Si system allowed an option for pediatric cases with an 8.5-mm camera and 5-mm instruments, which decreased in comparison with a 12-mm camera and 8-mm instruments of the standard option (8). Moreover, it is necessary to consider that the robot system is not available in all centers, and it presents higher costs than OP.

From 2003 to 2015, the rate of RALP augmented by 29% annually. However, most of these cases were children and adolescents. RALP was 40% among these patients in 2015. Differently, 85% of infants were still treated with OP (8). Many authors still prefer to perform an OP in infants. In these cases, the surgical procedure may be done by a mini-incision, avoiding muscle splitting, with reduced postoperative pain, fast patient recovery, and good aesthetic result. Therefore, the role of MIS in infants is still controversial: Tanaka et al. have reported that the benefits of LAP were evident only in older children (27).

The incidence of complications such as bleeding, UTI, or urinary leakage was similar between OP (10.0%) and MIS (10.9%). Looking specifically at MIS procedures, Bansal et al. (26) reported a higher complication rate in RALP (33.3%) than OP (6.6%), whereas Chandrakhaseram et al. in their meta-analysis focusing on infants (7) reported more complications in RALP (16.2%) than LAP (9.3%).

In a recent meta-analysis, the success of LAP and RALP in infants was found to be similar, with RALP having more OT duration and complications than LAP (21). Analyzing the KID database, Liu et al. evidenced that the use of MIS in children has gradually boosted from 0.3% in 2000 to 11.7% in 2009, with RALP representing 82% of these cases (40). Nevertheless, studies have reported outcomes on a small number of infants (13). In fact, in the present systematic review and meta-analysis, the included studies comparing OP and MIS techniques were relatively scarce: all papers were published between 2008 and 2022. Among 3,145 pyeloplasties, only 9.1% were performed with MIS, with an equal distribution between LAP (50.7%) and RALP (49.3%).

Finally, different from the previous report, where the follow-up was longer for the traditional OP (25, 26, 32), this meta-analysis presented no statistically significant differences between OP and MIS in terms of length of postoperative follow-up. This result highlights that MIS has already been used for a sufficient time to compare the outcome of both techniques.

### Limitation of the study

There are several limitations of the present study. As reported above, all but one studies were retrospective, which may lead to select bias. None of the papers provided sample size calculations. As expected, a blinded evaluation of objective endpoints was not possible. Moreover, the outcomes of MIS were strictly dependent on procedural volume. High-volume centers presented perioperative outcomes that were equivalent to or better than those of OP, different from low-volume centers (28). Furthermore, none of the studies have reported with regard to the loss of follow-up. As a consequence, in our meta-analysis, none of the studies reached the gold standard cutoff on MINORS of 19.8 out of 24 (Table 3).

**Table 3:** Risk of bias assessment for individual studies using the methodological index for nonrandomized studies (MINORS) (18).

Item	Masieri (1)	Andolfi (13)	Neheman (22)	Garcìa-Aparicio (24)		Tong (25)	Bansal (26)	Tanaka (27)	Rague (30)	Dangle (11)
1. A clearly stated aim	2	2	2	2		2	2	2	2	2
2. Inclusion of consecutive patients	2	2	2	2		2	2	2	2	2
3. Prospective collection of data	0	2	0	0		0	0	0	0	0
4. Endpoints appropriate to the aim of the study	2	2	2	2		2	2	2	2	2
5. Unbiased assessment of the study endpoint	0	0	0	0		0	0	0	0	0
6. Follow-up period appropriate to the aim of the study	1	1	1	0		1	1	1	1	1
7. Loss to follow-up less than 5%	0	0	0	0		2	0	0	0	0
8. Prospective calculation of the study size	0	0	0	0		0	0	0	0	0
9. An adequate control group	2	2	2	2		2	2	2	2	2
10. Contemporary groups	2	2	2	2		2	2	2	2	2
11. Baseline equivalence of groups	1	1	2	2		2	1	2	1	1
12. Adequate statistical analyses	2	2	2	2		2	2	2	2	2
Total score	14	16	15	14		17	14	15	14	14

**0 **= not reported; **1 **= reported but inadequate; **2 **= reported and adequate.

Validated “gold standard” cut-off: 19.8.

According to the GRADE methodology, the quality of evidence of the meta-analysis was low regarding the length of hospital stay, the incidence of postoperative complications, and the failure of the surgical procedure (Table 4). Both the reduced number of MIS infants and the considerable heterogeneity of the data could generate possible bias.

**Table 4:** GRADE evidence profile (19) for the present meta-analysis.

Quality assessment							No. of patients		Effect		Quality
No. of studies	Study design	Risk of bias	Inconsistency	Indirectness	Imprecision	Other considera-tions	Cases	Controls	Relative (95% CI)	Absolute (95% CI)	
Operative time between MIS and OP in infants							MIS	OP			
7	OS	Moderate^a^	Substantial	Not serious	Serious^b^	None	225	332	—	MD 18.19 lower (from 28.35 to 8.04 lower)	⊗OOO VERY LOW
Length of hospital stay between MIS and OP in infants							MIS	OP			
9	OS	Moderate^a^	Substantial	Not serious	Serious^b^	None	286	2,898	—	MD 0.76 higher (from 0.16 to 1.36 higher)	⊗⊗OO LOW
Incidence of complications between MIS and OP in infants							MIS	OP			
8	OS	Moderate^a^	Moderate	Not serious	Serious^b^	None	27/248 (10.9%)	32/319 (10.0%)	RR 0.95 (0.45, 2.01)	9 more per 1,000 (from 99 less to 182 more)	⊗⊗OO LOW
Incidence of failure between MIS and OP in infants							MIS	OP			
7	OS	Moderate^a^	Low	Not serious	Serious^b^	None	10/238 (4.2%)	116/308 (5.2%)	RR 1.28 (0.58, 2.82)	10 fewer per 1,000 (from 15 fewer to 65 more)	⊗⊗OO LOW

MIS, minimally invasive surgery; OP, open pyeloplasty.

GRADE Working Group grades of evidence.

High quality: Further research is very unlikely to change our confidence in the estimate of effect.

Moderate quality: Further research is likely to have an important impact on our confidence in the estimate of effect and may change the estimate.

Low quality: Further research is very likely to have an important impact on our confidence in the estimate of effect and is likely to change the estimate.

Very low quality: We are very uncertain about the estimate.

^a^ Bias due to possible confounding.

^b^ OIS not met.

However, when assessed in duplicate by two authors (DDR and MEM) using A Measurement Tool to Assess Systematic Reviews (AMSTAR) (43), the present study received an honest score (Supplementary material S2).

The PRISMA checklist was finally fulfilled (Supplementary material S3).

## Conclusion

The present systematic review and meta-analysis showed that MIS seemed a safe and effective procedure for surgically treating UPJO in infants. MIS procedures present similar outcomes in terms of success rate and postoperative complications to OP, in front of a shortened length of hospital stay. In the current practice, LAP and RALP may be used as an alternative to the traditional open technique, but their several limitations must be recognized.

However, more high-quality data from well-designed randomized control trials and sufficient adjustment for volume outcome are necessary to indicate the feasibility and safety of MIS in infants compared with OP. Until then, in our opinion, only experienced surgeons should perform MIS procedures in infants, with appropriate counseling with the family to evaluate the benefits and limitations of each technique.

## Data Availability

The raw data supporting the conclusions of this article will be made available by the authors without undue reservation.
