# Supplementary material for: Ureteropelvic junction obstruction in infants: Open or minimally invasive surgery? A systematic review and meta-analysis
Source: Front Pediatr. 2022 Nov 23;10:1052440. doi: 10.3389/fped.2022.1052440 (PMC9727311; doi:10.3389/fped.2022.1052440)
Supplement: Supplementary file 1 [file Table1.doc]

**Supplementary file 1:** Search strategy.

**PubMed/MEDLINE**

1. (pyeloplast*adj2).mp.
2. (infant*adj2).mp.
3. 1 AND 2

**Scopus**

TITLE-ABS-KEY ( ( pyeloplasty ) AND ( infants ) )

**Cochrane Collaboration**

1. MeSH descriptor : pyeloplasty, infants
2. Explode all trees

**Web of Science**

TOPIC ( ( pyeloplasty ) AND ( infants ) )
